# Correlation between Clinical and Histopathological Diagnoses in Oral Cavity Lesions: A 12-Year Retrospective Study

**DOI:** 10.1155/2022/1016495

**Published:** 2022-05-14

**Authors:** Golnoush Farzinnia, Mehdi Sasannia, Shima Torabi, Fahimeh Rezazadeh, Alireza Ranjbaran, Azita Azad

**Affiliations:** ^1^Oral and Dental Disease Research Center, School of Dentistry, Shiraz University of Medical Sciences, Shiraz, Iran; ^2^Student Research Committee, Shiraz University of Medical Sciences, Shiraz, Iran; ^3^Department of Oral & Maxillofacial Pathology, School of Dentistry, Shiraz University of Medical Sciences, Shiraz, Iran; ^4^Oral and Dental Disease Research Center, Department of Oral & Maxillofacial Medicine, School of Dentistry, Shiraz University of Medical Sciences, Shiraz, Iran

## Abstract

**Objective:**

Proper diagnosis plays a key role in the treatment and prognosis of all diseases. Although histopathological diagnosis is still known as the gold standard, final diagnosis becomes difficult unless precise clinical descriptions are obtained. So, this study aimed to evaluate the concordance of the clinical and histopathological diagnoses of all oral and maxillofacial biopsy specimens in a 12-year duration.

**Materials and Methods:**

Archive files and clinical findings related to 3001 patients who had been referred to the Department of Oral Pathology during a 12-year period were reviewed. The recorded information in files included age, sex, lesion's location, clinical and histopathological diagnoses, and specialty of dentists.

**Results:**

Out of 3001 cases included and reviewed in this study, 2167 cases (72.2%) were consistent between clinical and histopathologic diagnoses. Age, sex, and clinician's specialty were indicated to have no significant effect on diagnosis (*p* values = 0.520, 0.310, 0.281, respectively), but location and type of lesion affected that (*p* values = 0.040 and 0.022, respectively). In regard to location, the highest concordance of clinical and histopathologic diagnoses was observed in mouth floor lesions, and the lowest one was in gingival mucosa. In terms of lesion category, the highest and the lowest concordance rates belonged to white and red lesions and pigmented lesions, respectively.

**Conclusion:**

The results of the present study show that the consistency of clinical and histopathological diagnoses was three times more than their inconsistency, and the accuracy of the clinicians was largely acceptable.

## 1. Introduction 

The oral cavity is a complex area in the located in the head and neck regions and home to a diverse range of cysts, benign, and malignant salivary gland tumors, as well as odontogenic and nonodontogenic neoplasms [1, 2]. Both the diagnosis and treatment of oral cavity lesions are known as integral parts of oral health care [3]. Moreover, it is well understood that early detection and treatment of these lesions would greatly lead to the improvement of patients' survival rates and quality of life [4]. Although each oral lesion has different characteristics and clinical features aiding in diagnosis, clinical diagnosis errors occur due to the similarities in clinical presentations, lack of precise definitions for these characteristics, incompatibility of the signs and symptoms in patients, and the presence of multiple manifestations for a lesion [5, 6]. Therefore, in order to minimize misdiagnoses and to achieve more accurate ones, it is necessary to consider the patients' chief complaints, medical and dental histories' records, clinical manifestations, imaging diagnostic techniques, and various tests like laboratory tests that include biopsies with microscopic evaluations and blood tests [6]. Histopathologic examination, which is known as the gold standard in diagnostic oral pathology, is used to confirm the clinical diagnosis [7]. However, pathologists may encounter uncertainty during performing the histological examination on lesions under some circumstances, because various lesions may exhibit comparable microscopic views. Thus, the clinical examination can be considered as an effective and important step for confirming pathology results and will also be quite useful in such situations [8]. Therefore, the initial clinical diagnosis made by clinicians must be accurate. Moreover, it should not miss any oral potentially malignant disorders (OPMDs) or malignant lesions, and a close collaboration between the clinician and the pathologist is required in this regard, in order to reach a definitive and right diagnosis [2]. Various studies have previously investigated the concordance of clinical and pathologic diagnoses, and as a result, they reported concordance rates of approximately between 50 and 80% [3, 5–8]. Due to the reported discrepancy in the concordance rates between clinical and histopathological diagnoses in numerous studies performed in various places, the present study aimed to determine the rate of discrepancy between clinical and histopathological diagnoses. This research was done on the patients admitted to Shiraz dentistry school with the hope that the obtained results would help identify weaknesses in the diagnosis of oral diseases and improve both diagnostic and treatment outcomes.

## 2. Materials and Methods

This study was performed in the Faculty of Dentistry, Shiraz University of Medical Sciences, in terms of all relevant principles of the Helsinki Declaration. All the included subjects signed informed consent forms, and the ethical approval was obtained from the ethics committee of Shiraz University of Medical Sciences, Shiraz (IR.SUMS.DENTAL.REC.1398.123).

In this retrospective study, all the oral lesions diagnosed between January 2006 and December 2018 were then extracted from the archives that existed in the Department of oral pathology. Clinical examinations have been performed and approved by oral medicine specialists and maxillofacial surgeons who had sufficient skills in this field. For the purpose of this study, the census method was firstly used to select the eligible subjects, and the exclusion criteria were as follows: records with inadequate information, biopsy samples without definite pathological reports, and lesions in which a clinical impression was not given. In the patients' records, the following data were available: demographic data (age and gender), location of the lesion (mandible, maxilla, palate, alveolar mucosa, buccal mucosa, labial mucosa, ventral surface of tongue, dorsal surface of tongue, lateral surfaces of tongue, floor of mouth, gingiva, and lip), clinician's specialty (oral medicine and oral surgeon), and the clinical and pathological diagnoses of the lesions. All the included cases were subdivided into the following five groups based on the clinical manifestations.

1. Ulcerative, vesicular, and bullous lesions
2. Red and white lesions
3. Pigmented lesions
4. Bone lesions, which were divided into either cystic or tumoral (benign/malignant) lesions
5. Exophytic soft tissue lesions, which were divided into either reactive/inflammatory or tumoral (benign/malignant) lesions

This classification of lesions was done according to the textbook of oral diseases (Burket's ORAL MEDICINE 12th edition) [9]. The histopathological criteria for the final pathological diagnosis of each lesion were based on the textbook of Oral and Maxillofacial Pathology [10]. Finally, the obtained samples with a similar diagnosis using both techniques were recorded as the concordance of clinical and pathological diagnoses.

The collected data from all groups were imported to Statistical Package for Social Sciences (SPSS) for Windows software, version 16.0 (SPSS Inc., Chicago, IL, USA). As well, descriptive statistics indices were used to calculate the absolute and relative frequencies of different lesions. The chi-square test was used to compare the categorical demographic variables among the groups. The confidence interval was set to 95%, and *p* < 0.05 was considered statistically significant.

## 3. Results

A total of 3001 clinical files were evaluated in the current study. In 2167 cases (72.2%), the clinical and pathological diagnoses were consistent.

### 3.1. Age and Sex

Among all the biopsied cases, 1432 (47.7%) men and 1569 (52.3%) women were included. Moreover, 1058 male (73.9%) and 1109 (70.7%) female subjects had consistent diagnoses between clinical and pathological. In addition, 2708 (93.2%) cases were in the second decade of their life, so they were the most prevalent cases. After the tenth, ninth, and eighth decades (with a total of 9 cases), the sixth decade had the most frequent clinical and histological concordance (78%), and the fifth decade had the least (51.6%) (Table 1). Of note, there was no significant relationship between patients' sex and age and concordance of clinical and histopathologic diagnoses (*P* values = 0.310 and 0.520, respectively).

### 3.2. Clinician's Specialty

Among the total subjects, 1428 (47.6%) cases were referred from oral and maxillofacial medicine, and 1573 cases (52.4%) were from the oral and maxillofacial surgery department. As well, 75% of the referrals from the medicine department were consistent between clinical diagnosis and pathology, the rate of which was 69.7% for the surgery department. No significant relationship was found between the clinician's specialty and concordance of clinical and histopathologic diagnoses (*P* value = 0.281).

### 3.3. Location

Of the 12 documented biopsy sites, the mandible was observed to be the most common one accounting for 770 (25.6%) cases, followed by the floor of the mouth accounting for the least biopsied sites with 27 biopsies and with the highest rate of concordance (85.2%). Notably, the minimum rate of concordance was found to be related to gingival lesions (66.1%). A significant relationship was also found between the lesion's site and concordance of clinical and histological diagnoses (*P* value = 0.040) (Table 2).

### 3.4. Categories of Lesions

As mentioned earlier, all the cases included in this study were divided into 5 categories (Table 3). Exophytic lesions that were observed in 44.1% (*n* = 1326) cases were the most common category of lesions. Biopsy of pigmented lesions was the least type by detecting only in 1.1% (*n* = 34) cases. Red and white lesions accounted for the highest rate of concordance (86.1%) and the least rate belonged to pigmented lesions (47.1%). As well, a significant relationship was found between the type of lesion and concordance of clinical and histological diagnoses (*P* value = 0.022). In this regard, the frequency and concordance rate of lesions in each category are shown in Table 4.

## 4. Discussion

In this study, the rate of concordance between the two clinical and histopathological diagnoses was examined, along with the prevalence of each biopsied lesion submitted to the Department of Oral Pathology, Shiraz dentistry school. Accordingly, these considerations are valuable for improving the existing knowledge about the perception and behavior of dentists and dental students regarding the necessity of performing the histopathological examination. In the present study, the rate of clinicopathological concordance was obtained as 72.2%, which is similar to those obtained in studies by Saravani et al. [11] and Emamverdizadeh et al. [8] who calculated the overall concordance rate as 70.1% and 72.3%, respectively. However, our concordance rate was low when compared to studies conducted by Tatli et al. [2] and Forman et al. [12] (93.3% and 94.4%, respectively). This can be accrued to more sample size and the diversity of lesions in our study. In a study by Soyele et al. [13], clinicopathological reports of 592 biopsied cases during the period of 2008–2017 were retrieved and then analyzed. Accordingly, they recorded the concordance rate as 54.6%, which was similar to the results of Poudel et al.'s study [7] (54.6%). These discrepancies could be due to remarkable differences in these studies' methodologies such as the clinicians' and the pathologists' skills, the accuracy of biopsy, sample size, and conditions under which the specimens were transferred to the laboratory.

Based on the fact that some lesions occur more frequently in one sex or at certain ages, so it can be said that age or sex can be considered as one of the influential factors in making a better differential diagnosis. However, in the present study, no significant relationship was observed between concordance rate and sex or age. These findings are in line with those of Saravani et al.'s study [11]. However, in Forman et al.'s research [12], age was found to be significantly associated with accuracy between clinical and histological diagnoses. Furthermore, in the current study, the highest concordance rate after the tenth, ninth, and eighth decades (with a total of 9 cases in almost 3000 cases) was observed in the sixth decade of life, which is almost consistent with other similar reports, demonstrating that the highest percentage of concordance rate was observed in the seventh decade and older age [13–17]. The reason for the greater concordance rate between clinical and pathological diagnoses in this age group may possibly be the loss of teeth, thereby the reduced number of odontogenic lesions and irritation associated with them. Another reason might be the exclusion of lesions developing in children or young adults. Moreover, a slight increase might be found in some specific lesions such as denture-related lesions and other prevalent lesions, which consequently makes a correct diagnosis of lesions easier [11, 14]. Despite the results of the present study, two previous studies [12, 13] have also observed a higher concordance index in women, while another study [2] has reported slightly higher discordance rates for the female patients' lesions compared to the male patients' ones.

Similar to the current study, Saravani et al. [11] have also found no relationship between concordance of clinical and histopathological diagnoses and the clinician's specialty. However, in the study by Foroughi et al. [18], the highest and lowest concordance rates between clinical and pathological diagnoses were achieved by oral medicine specialists (98%) and general dentists (71%), respectively. The current study indicated that a significant relationship exists between the lesion's site and concordance of clinical and histological diagnoses. Gingival lesions and floor of mouth both had the minimum and maximum rates of concordance in the current study, respectively. Correspondingly, this finding may be due to the fact that several oral diseases have the same clinical manifestations in gingiva; for example, desquamative gingivitis can be seen in either ulcerative and vesiculobullous or white and red lesions, so it is not clinically distinguishable among these types of diseases. However, Foroughi et al. [18] and Hashemipour et al. [16] in their studies reported the most concordance rate of clinical and histopathological diagnoses in the gingiva. Furthermore, the lowest concordance rate was observed on the floor of the mouth, as reported in Hashemipour et al. and Saravani et al.'s studies [11, 16]. These contradictory findings in these studies may be due to variations in the sample size and the clinicians' knowledge and experiences.

The present study is unique as it, for the first time, examined a large number of studied biopsy samples and then classified all lesions into 5 categories of ulcerative, white and red, pigmented, exophytic, and bone lesions, which include almost all types of oral lesions while other studies have mainly focused only on few specific lesions and a specific group [19–22]. According to the results of the current study, a statistically significant relationship exists between the concordance rate of the histopathological and clinical diagnoses and the type of lesions. Accordingly, this finding is in line with the results of the study by Saravani et al. [11] who found a significant relationship between the type of lesion (either neoplastic or nonneoplastic) and clinicopathological concordance. In this study, out of 5 general categories of lesions, the highest prevalence belonged to exophytic lesions, white and red lesions had the highest concordance rate, and pigmented lesions had the lowest rate. In white and red lesions, oral lichen planus was the most commonly observed lesion, and it also had the highest percentage of concordance (88.6%). Similarly, Fattahi et al. [14] in their study found the highest percentage of concordance for lichen planus (100%), and in another study, Goyal et al. [21] found the lichen planus as the most common lesion in oral mucosal lesions with the clinicopathological concordance rate of 91.4%.

As stated earlier, several investigations conducted on the concordance of clinical and pathological diagnoses have reported varying concordance rates as their results. Since the correct clinical or pathological diagnosis of lesions is closely linked to both the knowledge and educational level of clinicians, it is critical to redesign students' educational programs totally and then improve them. In order to avoid diagnostic errors, physicians and dentists should also take thorough histories of patients and then transmit them to pathologists, besides following proper and standard procedures when taking biopsies.

## 5. Conclusion

The results of the present study indicate that there is a concordance between the clinical and pathological diagnoses of the lesions in more than 70% of cases, but unfortunately, inconsistency still exists regarding some lesions, which is not negligible. So, it should be noted that the clinicopathological concordance rate will never reach 100%, because there are lesions that have the same clinical appearance and different histopathology, and in many of them, the definitive diagnosis is still based on the histopathological results. Therefore, to avoid misdiagnosis and improper treatment, all dental specialists should be informed and aware of the importance of sending all excised specimens for performing histological investigations.

## Figures and Tables

**Table 1:** Concordance rate of clinical and histopathologic diagnosis based on age ranges.

Decade (age ranges)	Total cases	Concordance N (%)	*P* value
1 (0–9)	4	2 (50%)	0.520
2 (10–19)	2797	2038 (72.6%)	
3 (20–29)	54	37 (68.5%)	
4 (30–39)	47	32 (68.1)	
5 (40–49)	31	16 (51.6%)	
6 (50–59)	41	32 (78%)	
7 (60–69)	18	12 (66.7%)	
8 (70–79)	6	5 (83.3%)	
9 (80–89)	2	2 (100%)	
10 (90–99)	1	1 (100%)	

**Table 2:** Concordance rate of clinical and histopathologic diagnosis based on location.

Site of lesion	Total cases	Concordance *N* (%)	*P* value
Mandible	770	516 (67%)	0.040
Maxilla	495	346 (69.9%)	
Palate	122	83 (68%)	
Alveolar mucosa	80	56 (70%)	
Buccal mucosa	479	399 (83.3%)	
Labial mucosa	151	124 (82.1%)	
Ventral surface of tongue	38	27 (71%)	
Dorsal surface of tongue	100	77 (77%)	
Lateral surfaces of tongue	190	130 (68.4%)	
Floor of mouth	27	23 (85.2%)	
Gingiva	410	271 (66.1%)	
Lip	139	115 (82.7%)	

**Table 3:** Concordance rate of clinical and histopathologic diagnosis based on the type of lesions.

Category of lesion	Total cases	Concordance *N* (%)	*P* value
Ulcerative, vesicular, and bullous lesions	75	42 (56%)	0.022
Red and white lesions	519	447 (86.1%)	
Pigmented lesions	34	16 (47.1%)	
Exophytic soft tissue lesions	1326	893 (67.3%)	
Bone lesions	1047	769 (73.5%)	

**Table 4:** Frequency and concordance rate of clinical and histopathologic diagnosis in each category of lesions.

Lesion	Total cases	Concordance *N* (%)
Ulcerative, vesicular, and bullous lesions		
Pemphigus vulgaris	46	34 (73.9%)
Pemphigoid	15	3 (20%)
Eosinophilic ulcers of tongue	9	3 (33.3%)
Traumatic ulcers	3	0 (0%)
Recurrent aphthous stomatitis	1	1 (100%)
Erythema multiform	1	1 (100%)

White and red lesions		
Lichen planus	449	398 (88.6%)
Leukoplakia	63	49 (77.8%)
Oral erythroplakia	4	0 (0%)
Lupus erythematosus	2	0 (0%)
Hairy leukoplakia	1	0 (0%)

Pigmented lesions		
Oral/Labial melanotic macule	14	11 (78.6%)
Inflammatory hyperpigmentation	6	0 (0%)
Melanocytic nevus	6	2 (33.3%)
Oral melanoacanthoma	4	2 (50%)
Malignant melanoma	3	1 (33.3%)
Melanosis	1	0 (0%)

Exophytic soft tissue lesions^1^		
Reactive/Inflammatory lesions	1100	742 (67.4%)
Fibroma	361	247 (68.4%)
Pyogenic granuloma	252	137 (54.4%)
Mucocele	174	160 (91.9%)
Epulis fissuratum	125	109 (87.2%)
Peripheral giant cell granuloma	124	57 (46%)
Peripheral odontogenic fibroma	38	18 (47.4%)
Epulis granulomatosa	14	9 (64.3%)
Neurofibroma	12	5 (41.7%)
Benign tumoral lesions	83	43 (52%)
Oral papilloma	50	33 (66%)
Pleomorphic adenoma	15	5 (33.3%)
Lipoma	5	0 (0%)
Schwannoma	5	1 (20%)
Hemangioma	4	0 (0%)
Traumatic neuroma	2	2 (100%)
Lymphangioma	1	1 (100%)
Basal cell adenoma	1	1 (100%)
Malignant tumoral lesions	143	108 (75.5%)
Squamous cell carcinoma	132	104 (78.8%)
Basal cell carcinoma	3	1 (33.3%)
Lymphoma	3	0 (0%)
Mucoepidermoid carcinoma	3	1 (33.3%)
Adenoid cystic carcinoma	2	2 (100%)

Bone lesions^2^		
Cystic lesions	861	669 (77.7%)
Radicular cyst	420	358 (85.2%)
Odontogenic keratocyst	184	115 (62.5%)
Dentigerous cyst	173	134 (77.4%)
Residual cyst	54	36 (66.7%)
Nasopalatine canal cyst	18	18 (100%)
Traumatic bone cyst	11	7 (63.6%)
Aneurysmal bone cyst	1	1 (100%)
Benign tumoral lesions	133	71 (53.4%)
Central giant cell granuloma	50	26 (52%)
Ameloblastoma	33	16 (48.5%)
Odontoma	15	13 (86.7%)
Osteoma	11	7 (63.6%)
Cementoblastoma	7	4 (57.1%)
Adenomatoid odontogenic tumor	7	1 (14.3%)
Central odontogenic fibroma	1	1 (100%)
Odontogenic myxoma	7	3 (42.9%)
Ameloblastic fibroma	1	0 (0%)
Malignant tumoral lesions	14	9 (64.3%)
Osteosarcoma	11	6 (54.5%)
Fibrosarcoma	2	2 (100%)
Chondrosarcoma	1	1 (100%)
Other^*∗*^	39	20 (51.2%)

1. Exophytic lesions were subdivided into two subgroups: reactive/inflammatory and tumoral lesions (malignant and benign tumors). 2. Bone lesions were subdivided into two subgroups: cystic and tumoral lesions (malignant and benign tumors). ^*∗*^Bone samples that were not included in either cystic or tumoral lesion were named “other”. This category includes developmental lesions of bone (fibrous dysplasia, ossifying fibroma, and periapical cemento-osseous dysplasia).

## Data Availability

The datasets analyzed during the current study are available from the corresponding author on reasonable request.
